# What Is a Consultant's Fee?

**Published:** 1898-12-31

**Authors:** 


### WHAT IS A CONSULTANT'S FEE?

If "the modest guinea now regarded as the minimum fee of all self-respecting consultants" were really the true minimum, and if it were the fact that consultant advice were never given for less, it would, we think, hardly have entered into the brain of Mr. Arthur Chamberlain to ask consultants to sell their services "in bulk," as the saying is, and allow the purchaser to retail them again to the outside public at less than half the lowest ordinary retail price. It is the belief, not altogether unfounded, that although the physician will take the guinea when it is offered he will quite willingly take much less if some plausible excuse is put forward, which makes the consultants appear fair game, and leads to the devising of such schemes as the one which was recently put forward in London, a scheme which did at least have some little grace of charity about it, and this more barefaced plan, which appears to be entirely devoid of even such excuse. The fact is that a good deal of that old system of "advice gratis" which the general practitioner has long ago learned to regard with disfavour still lingers around consulting work, and it is not surprising that outsiders should attempt to organise for the benefit of the general public a system of cheap consultation which they believe to be even now in operation, and available for all who can make up plausible little tales. If, then, it is true that the guinea by no means represents the minimum fee accepted by many who are doing consulting work, does it not seem hard, nay, does it not seem a little mean and underhand, that professional custom should debar them, if they would be considered "self-respecting consultants," from openly naming the fee that they are willing to take, and should condemn them to wrap up and disguise these cheaper fees by inventing little subterfuges, such as seeing a patient several times for a guinea?

